# Palliative care for nursing home patients with dementia: service evaluation and risk factors of mortality

**DOI:** 10.1186/s12904-020-00627-9

**Published:** 2020-08-12

**Authors:** Chih-Pang Chu, Cho-Yin Huang, Chian-Jue Kuo, Ying-Yeh Chen, Chun-Tse Chen, Tien-Wei Yang, Hsing-Cheng Liu

**Affiliations:** 1 Department of General Psychiatry, Songde Branch (Taipei City Psychiatric Center), Taipei City Hospital, 309 Sung-Te Road, Taipei, 110 Taiwan; 2 Department of Psychiatry, School of Medicine, College of Medicine, Taipei Medical University, Taipei, Taiwan; 3 Psychiatric Research Center, Taipei Medical University Hospital, Taipei, Taiwan; 4 Department of Public Health, National Yang-Ming University, Taipei, Taiwan; 5 University of Taipei, Taipei, Taiwan

**Keywords:** Palliative care, Dementia, Risk factor, Mortality

## Abstract

**Background:**

Difficulties in prognostication are common deterrents to palliative care among dementia patients. This study aimed to evaluate the effectiveness of palliative care in reducing the extent of utilization of medical services and the potential risk factors of mortality among dementia patients receiving palliative care.

**Methods:**

We surveyed dementia patients involved in a palliative care program at a long-term care facility in Taipei, Taiwan. We enrolled 57 patients with advanced dementia (clinical dementia rating ≥ 5 or functional assessment staging test stage 7b). We then compared the extent of their utilization of medical services before and after the provision of palliative care. Based on multivariable logistic regression, we identified potential risk factors before and after the provision of palliative care associated with 6-month mortality.

**Results:**

The utilization of medical services was significantly lower among dementia patients after the provision of palliative care than before, including visits to medical departments (*p* < 0.001), medications prescribed (*p* < 0.001), frequency of hospitalization (*p* < 0.001), and visits to the emergency room (*p* < 0.001). Moreover, patients dying within 6 months after the palliative care program had a slightly but not significantly higher number of admissions before receiving hospice care (*p* = 0.058) on univariate analysis. However, no significant differences were observed in multivariate analysis.

**Conclusions:**

The provision of palliative care to dementia patients reduces the extent of utilization of medical services. However, further studies with larger patient cohorts are required to stratify the potential risk factors of mortality in this patient group.

## Background

Dementia is a neurodegenerative disorder that leads to a decline in multiple brain functions including memory, language, and balance and motor functions [1, 2]. Individuals with dementia generally need complex long-term care involving a multidisciplinary approach and integration of medical resources [3]. The prevalence of dementia has increased in Western [1, 3, 4] and Eastern populations [5] owing to aging populations, with a marked effect on nationwide healthcare systems and a considerable socioeconomic burden [6].

Given that dementia is a progressive and irreversible disease without any treatments for cure, implementing palliative care programs for patients with dementia has been considered an appropriate strategy [7, 8]. Palliative care programs target patients at a high mortality risk due to advanced-stage diseases and are designed to relieve suffering and improve quality of life by reducing unnecessary or aggressive therapies [9]. Such a caregiving approach has been more extensively used among cancer patients than among dementia patients [1, 10]. Insufficient insights into the terminal nature of dementia among caregivers and some medical professionals have limited the provision of palliative care to dementia patients [1, 11]. Furthermore, the unpredictability of the disease course [1, 2, 10]; difficulties with prognostication [10, 12]; and lack of professional policies, guidelines [9], or funding [12] are common deterrents to the provision of palliative care among dementia patients.

Some evidence suggests an increasing trend in the utilization of palliative care among dementia patients [10, 13]. For example, in the United States, enrollment of dementia patients to palliative care increased from < 1% to approximately 15% from 1995 to 2014 [10]. Another US survey using national data from Medicare revealed that > 50% of nursing home patients receiving palliative services were diagnosed with dementia in 2004 and 2009 [14]; moreover, the primary expenditures for palliative care among dementia patients are the use of inpatient hospital care and treatment [15]. However, compared with patients with other clinical conditions, end-stage dementia patients often receive suboptimal palliative care [1, 12]. The lack of highly predictable clinical variables in estimating the survival time of dementia patients has caused difficulty among caregivers and clinicians in determining the appropriate timing for deploying end-of-life management when providing care to these patients [7, 12]. Consequently, dementia patients potentially have higher chances of receiving aggressive intervention during their progressive functional decline and as they experience intermittent health conditions such as infections, dehydration, delirium, or falls [6, 10]. Furthermore, previous studies [16, 17] have reported an increasing trend in the proportion of dementia patients dying in nursing homes; however, a substantial proportion of such patients still die in hospitals, thus potentially reflecting the unnecessary utilization of medical services at the end stage of life and that the concept of palliative care in dementia has not been fully adopted in the medical care community.

Limited evidence has successfully identified mortality risk factors among advanced dementia patients receiving palliative care [12, 18]. Furthermore, evidence regarding the effectiveness of palliative care in reducing the utilization of medical services for dementia patients is inconsistent and inconclusive [6]. By analyzing data on dementia patients receiving palliative care in a nursing home in Taipei, Taiwan, we evaluated the effect of the provision of palliative care among terminal dementia patients by comparing the extent of utilization of medical services before and after a palliative care program. Furthermore, we evaluated the potential mortality risk factors among dementia patients receiving palliative care.

## Methods

### Data sources

In July 2014, a palliative care program was initiated in one nursing home in Taipei, Taiwan, by the team from the Taipei City Psychiatric Center (TCPC; Songde branch), Taipei City Hospital. Before 2014, nursing homes in Taiwan rarely provided palliative care; most of these services were provided in hospitals. TCPC has provided an intervention that integrates palliative care in nursing homes since July 2014 called the TCPC-Nursing Home Palliative Care Program. The program is operated by a multidisciplinary team from TCPC that includes board-certified neurologists, psychiatrists, psychiatric nurses, social workers, pharmacists, and clinical psychologists. In integrating the palliative care program, the team has collaborated well with the nursing staff in the nursing home. The inclusion criteria for this care program were determined in accordance with the regulations of Taiwan’s National Health Insurance program. The clinical conditions of potential patients considered for enrollment in this program are carefully evaluated by the neurologists or psychiatrists in the team. The participants (nursing home inhabitants) with terminal cancer; advanced dementia (clinical dementia rating ≥ 5 or functional assessment staging test [FAST] stage 7b); severe neurodegenerative disease other than dementia; or those with severe lung, liver, or heart disease are eligible for enrollment in this program.

The provision of care to late-stage dementia patients who are candidates for the palliative care program can be burdensome and of potentially limited clinical benefits owing to advanced cognitive and functional impairment in these patients [14]. This necessitated a transition in health care provision from futile or burdensome treatment to palliative care. Because dementia is a progressive and irreversible disorder without any cure, the implementation of the palliative care program entails several tasks, including the provision of adequate information to the families of the patients through family meetings and assisting the family members in signing a contract agreeing to palliative care and to entry into palliative care. Family meetings are held with the families of patients with terminal diseases to obtain their consent.

In this palliative care program, our program provides regular clinical services twice per week in the nursing home. These services comprise the prescription of drugs, evaluation of food intake, and the use of NG or Foley tubes. The multidisciplinary team is available 24 h per day to provide emergency consultations by an instant messaging software and an interim service would be arranged if the problem could not be resolved on line. The value of palliative care includes a reduction in the utilization of medical services (such as invasive treatment and unnecessary visits to the emergency room) and inconvenience to patients and their families. If a patient receiving palliative care dies, team members (particularly clinical psychologists) provide the post-mortem care to counsel the grieving family members, and the medical professionals (neurologists or psychiatrists) assist the family members in completing formalities related to the death certificate to facilitate the process of the funeral.

**Fig. 1:**
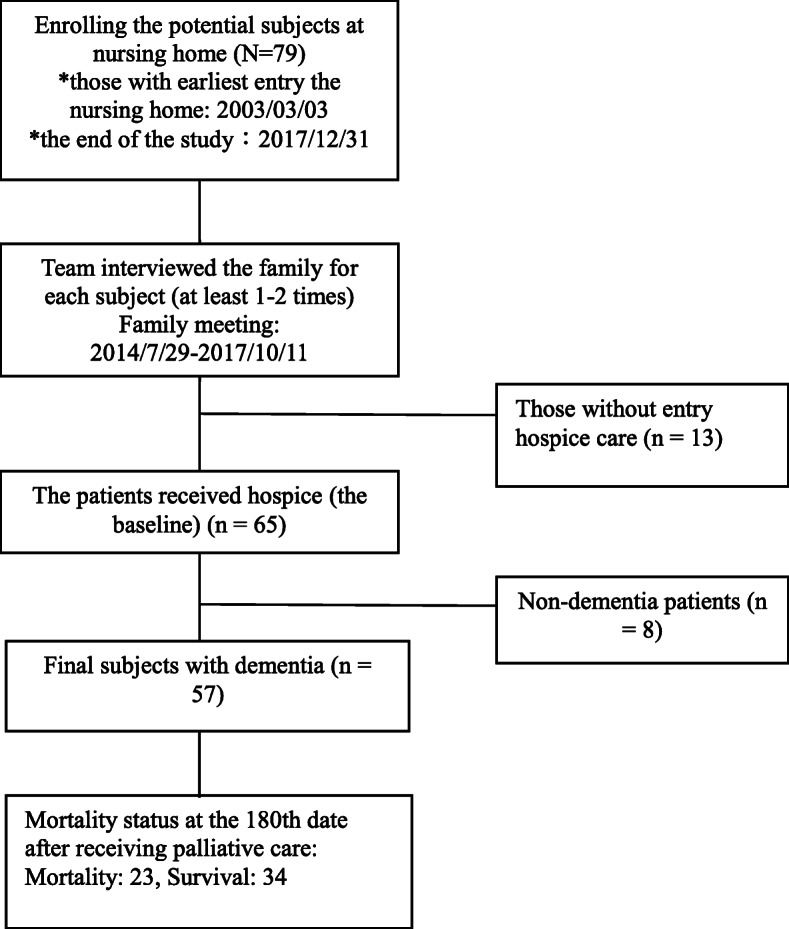
Flowchart of the study

By October 2017, 79 patients were enrolled in this program (Fig. 1), 65 agreed to undergo the palliative care program, and 57 had dementia and were included in this study for further assessment. The endpoint of the study was December 31, 2017. The Institutional Review Board of Taipei City Hospital approved the protocol for this study (TCHIRB-10707101-E). The survey used in the present study was developed for this study and has not previously been published elsewhere.

### Variables

We recorded the age, sex, physical comorbidities, and cognitive function of the patients upon admission to the nursing home and after entering palliative care. Furthermore, information regarding the utilization of health services, including the number of medical departments visited, number of prescribed medications, frequency of hospitalization, length of intensive care unit (ICU) admission, and the number of visits to the emergency department, were recorded before and after initiation of hospice care. We used the Barthel index [19] to evaluate functional abilities and the Mini-Mental Status Examination [20] to measure cognitive abilities. Mortality status on day 180 after the commencement of palliative care was examined. This study adopted the criteria for hospice eligibility in the Medicare system, requiring an estimated survival of less than 6 months [18, 21]. We described the survey in Additional file 1 and listed the study framework in the supplement e-Figure 1 (Please see Additional file 2).

### Statistical analysis

Descriptive analyses were performed to examine the demographic and clinical characteristics of dementia patients receiving palliative care. Moreover, we compared the average monthly indices for health care utilization before and after the provision of palliative care via a paired *t* test.

Compared with cancer patients receiving palliative care, dementia patients are more likely to remain in palliative care more than 6 months and require face-to-face recertification, per the regulations of the Centers for Medicare and Medicaid Services [22]. Accordingly, middle-term mortality was defined as patients dying within 180 d of receiving palliative care, and middle-term mortality was considered the endpoint to evaluate the potential risk factors regarding the extent of palliative care with such an endpoint. The potential risk factors that were taken as the clinical variables before palliative care, those upon receiving palliative care, and those after the provision of palliative care are summarized in Table 3. Univariate logistic regression analysis was performed to identify such variables associated with middle-term mortality within 180 d of receiving palliative care. Furthermore, the potential variables were subjected to multivariate logistic regression analysis with a backwards stepwise selection strategy, and those with *p* < 0.05 were retained in the final model.

Statistical significance was set at a two-tailed *p*-value < 0.05. All analyses were performed using SAS software, version 9.4 (SAS Institute Inc., Cary, NC, USA).

## Results

### Patient characteristics

The demographic and clinical characteristics of dementia patients receiving hospice care are listed in Table 1. In most cases, the patients’ children signed the contract for palliative care. Most of family members participated in family meetings once and then signed the contract for palliative care.

**Table 1:** Characteristics of dementia patients receiving palliative care

Characteristics	Men (*N* = 22)	Women (*N* = 35)
	***Mean (SD)***	***Mean (SD)***
Age at the receiving palliative care (y/o)	88.2 (5.6)	89.2 (5.2)
Duration from entry the nursing home to the receiving palliative care (years)	4.7 (4.1)	5.2 (4.2)
Duration from first family meeting to the entry of palliative care (days)	24.1 (78.1)	19.2 (45.8)
The person signed contract of palliative care	***N (%)***	***N (%)***
Couple	4 (18.2)	1 (2.9)
Child	14 (63.6)	30 (85.7)
Others	4 (18.2)	4 (11.4)
Family members participated Family meeting		
1–2	15 (68.2)	26 (71.1)
> 2	7 (31.8)	9 (28.9)
Times of Family Meeting		
1	25 (92.6)	30 (78.9)
2 (twice)	2 (7.4)	7 (18.4)

Information regarding the demographic data of the participants upon first receiving institutionalized hospice care are listed in supplement e-Table 1 (Please see Additional file 3). The clinical characteristics of patients upon admission to the nursing home and upon first entering institutionalized hospice care are listed in supplement e-Table 2 (Please see Additional file 4). Regarding the co-morbid conditions at the entry of nursing home, hypertension is the most prevalent one (57.8%), then stroke (38.6%), diabetes mellitus (26.3%), non-hypertension cardiovascular disease (22.8%).

### Service evaluation

Differences in the utilization of medical services among the dementia patients per month before and after the provision of palliative care are summarized in Table 2. The utilization of medical services, including visits to hospital departments (*p* < 0.001), medications prescribed (*p* < 0.001), frequency of hospitalization (*p* < 0.001), and visits to the emergency room (*p* < 0.001), was significantly lower after entry into palliative care.

**Table 2:** Differences in the utilization of medical services before and after the provision of palliative care

Characteristics (Average number per month)	Pre-palliative-care stage^b^ (*N* = 57)	Post-palliative-care stage^c^ (*N* = 57)	Statistics^a^
	***Mean (SD)***	***Mean (SD)***	
The departments visited	2.23 (0.96)	0.07 (0.26)	*P* < 0.001
Number of medications	7.84 (3.04)	5.39 (2.51)	*P* < 0.001
Number of hospitalizations	1.42 (1.45)	0.07 (0.32)	*P* < 0.001
Length of stay (days)	19.72 (22.74)	0.56 (3.21)	*P* < 0.001
Length of stay of ICU admissions (days)	1.49 (5.82)	0.00 (0.00)	*P* = 0.058
Number of visits on emergency department	0.39 (0.82)	0.02 (0.13)	*P* = 0.001

^a^Based on paired *t* test

^b^The average period of contribution in months (SD) in the prepalliative care stage: 10.53 (3.25)

^c^The average period of contribution in months (SD) in the postpalliative care stage: 9.07 (8.73)

### Factors associated with middle-term mortality

The potential factors associated with middle-term mortality 180 d after receiving palliative care are listed in Table 3. Univariate analyses revealed that patients dying within 180 d had more than two admissions before receiving hospice care with borderline statistical significance (*p* = 0.058) than did those who survived after 180 d. Otherwise, no significant risk factor was observed between patients surviving 180 d after palliative care and those who died within 180 d in terms of age upon entering hospice care, hospital departments visited per month before commencing palliative care, and medications prescribed per month before hospice care. Furthermore, no significant factor was retained in multivariate analysis.

**Table 3:** Before and after palliative care factors associated with mortality within 180 d of the provision of palliative care

Characteristics	Mortality within 6 months after palliative care (*N* = 23)	Survival at least 6 months after palliative care (*N* = 34)			
***Before palliative care (baseline)***	***N (%)***	***N (%)***	Odds ratio^a^	95% CI^a^	*P* value^a^
Age at receiving palliative care (y/o)					
≤ 85	5 (18.5)	6 (17.6)	Reference	–	–
86–90	8 (29.6)	15 (44.1)	0.64	0.15–2.77	0.550
> 90	10 (51.9)	13 (38.2)	0.92	0.22–3.92	0.914
Number of departments visited per month before palliative care					
1	2 (8.7)	9 (26.5)	Reference	–	–
2	13 (56.5)	17 (50.0)	3.44	0.63–18.72	0.153
> 2	8 (34.8)	8 (23.5)	4.50	0.73–27.74	0.105
Number of medications used per month before palliative care					
0–5	2 (8.7)	10 (29.4)	Reference	–	–
> 5	21 (91.3)	24 (70.6)	4.38	0.86–22.27	0.075
Number of admissions within 1 year before palliative care					
0	5 (21.7)	9 (26.5)	Reference	–	–
1	6 (26.1)	20 (58.8)	0.54	0.13–2.24	0.396
> 1	12 (52.2)	5 (14.7)	4.32	0.95–19.58	0.058
ICU admission within 1 year before palliative care					
Yes	4 (17.4)	1 (2.9)	Reference	–	–
No	19 (82.6)	33 (97.1)	0.14	0.02–1.38	0.093
Number for ER visits within 1 year before palliative care					
0	18 (78.3)	25 (73.5)	Reference	–	–
1–6	5 (21.7)	9 (26.5)	0.77	0.22–2.69	0.684
***At receiving palliative care***					
Cognitive impairment					
Severe	23 (100)	31 (91.2)	Reference	–	–
Moderate	0 (0)	2 (5.9)	0	0	1.000
Mild	0 (0)	1 (2.9)	0	0	0.999
Mobility					
Bedridden	22 (95.7)	26 (76.5)	Reference	–	–
Wheelchair	1 (4.3)	8 (23.5)	0.15	0.02–1.28	0.082
Intake					
NG tube feeding	20 (87.0)	28 (82.4)	Reference	–	–
Oral feeding	2 (8.6)	6 (17.6)	0.47	0.09–2.56	0.380
Gastroenteric tube feeding	1 (4.3)	0 (0)	2.26	0	1.000
Urination					
Diaper	13 (56.5)	20 (58.8)	Reference	–	–
urinary tube	9 (39.1)	11 (32.4)	1.26	0.41–3.87	0.688
Bladder fistula/Intestinal fistula	1 (4.3)	3 (8.8)	051	0.48–5.48	0.581
Respiratory function					
Spontaneous respiration	6 (26.1)	16 (47.1)	Reference	–	–
Tracheostomy	2 (8.7)	1 (2.9)	5.33	0.41–70.20	0.203
Need oxygen	15 (39.1)	17 (47.1)	2.35	0.73–7.56	0.151
Barthel Index, mean/SD	0 (0)	0 (0)	–	–	–
MMSE, mean [23]	9.95 (0.31)	9.87 (0.52)	0.82	0.20–3.35	0.778
***After palliative care***					
Number of departments visited per month after palliative care					
0	21 (91.3)	32 (94.1)	Reference	–	–
≥ 1	2 (8.7)	2 (5.9)	1.52	0.20–11.67	0.685
Number of medications use per month after palliative care					
0–5	10 (43.5)	22 (64.7)	Reference	–	–
> 5	13 (56.5)	12 (35.3)	2.38	0.81–7.04	0.116
Number of admissions after palliative care					
0	21 (91.3)	33 (97.1)	Reference	–	–
≥ 1	2 (8.7)	1 (2.9)	3.14	0.27–36.86	0.362
ICU admission after palliative care					
Nil	23 (100)	34 (100)			
Number of ER visits after palliative care					
0	23 (100)	33 (97.1)	Reference	–	–
1	0 (0)	1 (2.9)	0.00	0	1.000

*MMSE* Mini-Mental State Examination. ^a^Based on univariate logistic regression analysis

## Discussion

### Major findings

This study made two major findings. First, the utilization of medical services significantly decreased after the provision of palliative care to dementia patients compared with that before. Second, no significant mortality risk factors were identified among advanced dementia patients receiving palliative care.

### Characteristics of patients and service providers

All patients herein had a primary diagnosis of dementia, with hypertension and stroke being the most common comorbidities. Most of them did not have cancer. Moreover, most of these patients were bedridden, received tube feeding, and displayed deteriorated cognitive function upon entering the palliative care program compared with when they first entered the facility.

Regarding previous models used for delivering palliative care in nursing homes (NH), a study from the United States [24] reported three successful models for providing palliative care to nursing home residents, summarized as follows. Model 1: Palliative Care Consult Service—The palliative care service model involves external consultants on request of the NH medical director, the patient’s attending physician, or the NH director of nursing. Model 2: Nursing Home–Based Palliative Care—Nursing homes employ their own palliative care personnel. Model 3: Nursing Home–Palliative Care Partnerships—Well-integrated palliative care is provided in a nursing home [25]. The TCPC–nursing home palliative care model discussed herein is a hybrid of Model 1 and Model 3, wherein our medical professionals provide consultations, and regular care is provided at the nursing home. Our multidisciplinary model has enhanced access to palliative care for all nursing home residents. The team offers biweekly patient visits and monthly family meetings with their caregivers. Furthermore, medical team meetings involving all team members are held on a monthly basis to evaluate current treatment regimens.

### Service evaluation

Evidence regarding the effectiveness of palliative care in reducing the utilization of medical services among dementia patients is mixed. In accord with our finding regarding a reduction in the utilization of medical services after the provision of palliative care to terminal dementia patients, a study [26] reported that advanced dementia patients receiving palliative care at nursing homes in the United States received fewer medications, injections, feeding tubes, intravenous fluids, and medical services and they had a lower likelihood of hospital death than those who did not receive palliative care. Furthermore, a nationwide 5-year cohort study in Taiwan by Chen et al. [6] reported that palliative care is associated with a reduction in futile or burdensome treatments among dementia patients, except for tube feeding. However, subgroup analysis of dementia patients without a cancer diagnosis revealed that palliative intervention was associated only with low risks for cardiopulmonary resuscitation. By contrast, a randomized control trial in the United States involving hospitalized patients with advanced dementia receiving palliative intervention reported no effect on tube feeding, mechanical ventilation, and do-not-resuscitate decisions [27].

Some potential explanations regarding the discrepancies among these studies are the following. First, different comorbidities among the study patients may affect the correlation between the provision of palliative care and the utilization of medical services. Studies from Taiwan have reported different patterns in the use of lifesaving procedures depending on patient diagnoses (i.e., dementia, cancer, or both) [28]. For instance, patients with dementia had a higher frequency of admission to the hospital and the ICU and a longer duration of hospitalization than those with cancer [6, 29], indicating that a reduction in burdensome treatments among advanced dementia patients under palliative care among Taiwanese inpatients is more consistent among those with cancer. Furthermore, the proportion of cancer diagnoses was higher among the study patients than among dementia patients who did not receive palliative care [6]. In brief, having cancer could potentially be considered a protective factor against the use of burdensome treatment.

Second, the lack of guidelines regarding palliative care could result in differences in service patterns in palliative interventions among dementia patients in different studies, resulting in differences in the quality of care. Studies investigating staff perspectives on palliative care in dementia have reported that having limited guidelines that result in chaotic care is not uncommon from the perspective of clinical practitioners [1, 9]. Moreover, cultural differences regarding death and end-of-life issues could play a role in the service pattern of palliative care. For example, Taiwanese culture is based on principles of Confucianism, Taoism, and Buddhism, which have influenced Taiwanese individuals for thousands of years, particularly with respect to death [30]. Such cultural factors, including aspects of Confucian, Taoist, and Buddhist philosophies, influence the sense of autonomy of older residents, and they tend to rely on others to make decisions for them. Thus, when considering enrollment in the palliative care program, the team had to respect the family-oriented decision-making process of the older residents because it reflects a personal choice to make joint decisions with the family. The professionals would mediate communication between older residents and their families regarding concerns related to end-of-life care [30].

Furthermore, studies in Taiwan have reported that advanced dementia patients tend to more burdensome treatments during their last year of life in Eastern countries than in Western countries [29]. Moreover, discrepancies in the preferences of patients and caregivers regarding end-of-life management decisions are common [23, 31]. By contrast, studies in North America [32, 33] and Europe [34, 35] have reported a lower proportion (approximately 2–25%) of tube feeding among advanced dementia patients at the end stage of life compared with the proportion reported in studies conducted in Asia, where > 50% of dementia patients have been reported to be given enteral feeding [6, 35].

Our study investigated a rather homogenous group of patients without cancer and determined that by providing an explanation and psychoeducation on the terminal nature and disease course of dementia and the core value of palliative care, the quality of life of patients and their caregivers may be enhanced; moreover, the implementation of palliative care could significantly reduce burdensome treatments among dementia patients.

### Potential risk factors for the middle-term mortality

In this study, we could not identify significant mortality risk factors among advanced dementia patients receiving palliative care, which is consistent with previous studies. Lack of prognostic measures regarding the estimation of the survival duration among dementia patients deterred making decisions regarding the ideal timing for palliative care referral and the allocation of medical resources [1, 6, 10]. Importantly, under such circumstances, it is difficult for patients, families, and even clinicians to view dementia as a terminal condition. Studies on the survival of dementia patients have reported inconsistent findings with respect to survival duration, finding a mean or median survival duration in a range of approximately 3–10 years [7]. Studies have previously identified potential prognostic factors and mortality risk factors among dementia patients. For instance, a study in the United States [36] reported cancer, diabetes, the onset of organ failure, and mechanical ventilation as prognostic factors of hospice referral among dementia patients. Moreover, the U.S. National Hospice and Palliative Care Organization developed prognostic criteria for survival in dementia and as a tool to determine Medicare benefits for hospice care; according to their criteria, patients at advanced stages of the disorder (FAST stage 7c) with one or more nutritional (i.e., > 10% weight loss) or medical complications (i.e., recurrent sepsis, pressure sore) are more susceptible to death within 6 months [10]. However, the prognostic accuracy of these criteria have been questioned previously [18, 21]. Mitchell et al. further developed the Mortality Risk Index [21] as well as the Advanced Dementia Prognostic Tool [18], which used the dataset of nursing home residents in the United States to establish 12 items—age, sex, nursing home stay, shortness of breath, pressure ulcers, activities of daily living scale, bedfast most of day, insufficient oral intake, bowel incontinence, body mass index, recent weight loss, congestive heart failure—with modest predictability of death within 6 months among nursing home patients with dementia. However, the criteria validity among non-nursing home patients has not yet been examined. Thus far, there is a lack of consensus among studies worldwide regarding appropriate prognostic measures for dementia [1, 7], and our study further affirmed this observation. Univariate analysis herein revealed that more admissions before entering a hospice is a possible risk factor of 6-month mortality, potentially indicating that these patients already had multiple health conditions and were more susceptible to death. It is reasonable to conclude that patients with advanced disease stages and poor medical conditions have poor disease outcomes.

### Limitations

This study has several limitations. First, our patient cohort was relatively small and had moderate statistical power. This study preliminarily investigated the potential risk factors associated with the middle-term mortality endpoint in an exploratory manner. Hence, future studies with larger patient cohorts are required to investigate the predictors associated with such an endpoint.

Second, the study site was a nursing home located in a major city in Taiwan; hence, these results are not representative of the nationwide population. Nevertheless, unlike in previous studies, this study included a homogenous patient cohort with a primary diagnosis of dementia without complicated comorbidities under a standard treatment regimen.

## Conclusions

The present results have several clinical implications for professionals providing palliative care to advanced dementia patients. This evidence-based study found a significant reduction in the utilization of medical services, including visits to hospital departments, medications prescribed, number of hospitalizations, and visits to the emergency room, after the provision of palliative care to dementia patients. However, this study did not reveal any significant mortality risk factors among advanced dementia patients receiving palliative care, which precludes decisions regarding the ideal timing for palliative care referral and the allocation of medical resources. This study emphasizes the need for an integrated palliative care intervention for terminal dementia patients in nursing homes. Future studies with larger patient cohorts are required to stratify the potential prognostic factors of mortality in this patient group.

## Supplementary information

**Additional file 1.** The survey of the present study.

**Additional file 2: e-Figure 1.** Study framework.

**Additional file 3: e-Table 1.** Demographic data of the participants upon first receiving institutionalized palliative care.

**Additional file 4: e-Table 2.** Clinical characteristics of the patients upon entry into the nursing home and upon receiving institutionalized palliative care.

## Data Availability

The data collection transcripts are available on reasonable demand to the corresponding author.

## Abbreviations

FAST: Functional Assessment Staging Test
CDR: Clinical Dementia Rating Scale
NHI: National Health Insurance
MMSE: Mini-Mental Status Examination
ADEPT: Advanced Dementia Prognostic Tool
DNR: Do-not-resuscitate
ICU: Intensive care unit
