# Supplementary material for: Palliative care for nursing home patients with dementia: service evaluation and risk factors of mortality
Source: BMC Palliat Care. 2020 Aug 12;19:122. doi: 10.1186/s12904-020-00627-9 (PMC7425598; doi:10.1186/s12904-020-00627-9)
Supplement: Supplementary file 1 — Additional file 1. The survey of the present study. [file 12904_2020_627_MOESM1_ESM.doc]

**Additional 1.** The survey of the present study

**The Survey**

**Purpose of the research**

(1) To evaluate the effect of the provision of palliative care among terminal dementia patients by comparing the extent of utilization of medical services before and after a palliative care program.

(2) To evaluate the potential mortality risk factors among dementia patients receiving palliative care.

**Settings**

A nursing home in Taipei, Taiwan

**Intervention:**

Taipei City Psychiatric Center (TCPC) has provided an intervention that integrates palliative care in nursing homes since July 2014 called the TCPC-Nursing Home Palliative Care Program. The program is operated by a multidisciplinary team from TCPC that includes board-certified neurologists, psychiatrists, psychiatric nurses, social workers, pharmacists, and clinical psychologists. In integrating the palliative care program, the team has collaborated well with the nursing staff in the nursing home.

**Type of research design**

(1) Cross-section study design

(2) Follow up the status of 180-day mortality for each subject after receiving the palliative care

**Patient selection**

The participants (nursing home inhabitants) with advanced dementia (clinical dementia rating ≥5 or functional assessment staging test [FAST] stage 7b) with receiving the palliative care are eligible for enrollment in this program, from July 29, 2014 to October 11, 2017.

**Information collection**

Data on the following variables were collected by psychiatric nurses:

(1) Demographical variables: age, sex

(2) Information of family meeting

(3) Physical comorbidities

(4) Cognitive function of the patients upon admission to the nursing home and after entering palliative care.

(5) Utilization of health services: including the number of medical departments visited, number of prescribed medications, frequency of hospitalization, length of intensive care unit (ICU) admission, and the number of visits to the emergency department, were recorded before and after initiation of palliative care.

(6) Barthel index

(7) Mini-Mental Status Examination

(8) Mortality status on day 180 after the commencement of palliative care

**The form for data collection**

We listed the form for data collection as below:

The form for data collection

(1) Basic information

1. Research ID：__ __ __ __ __ __ __ __

2. Sex: □1.men □2.women

3. Birthday: ___/___/______

4. Education level：

| □ 1.postgraduate  □ 2.College  □ 3.Senior high school | □ 4.Junior high school  □ 5.Elementary school  □ 6.Illiterate  □ 9.Unknown |
| --- | --- |

The year of education: _____________

5. Marital status

| □ 1.married  □ 2.single  □ 3.divorce | □ 4.cohabited  □ 5.separated |
| --- | --- |

6. Spouse：□1.live □2.death

(2) Entry the nursing home

1. The date of entry the nursing home: ___/___/_____

2. Major caregiver

| □ 1.spouse  □ 2.son/daughter | □ 3.siblings  □ 4.others |
| --- | --- |

3. The diagnosis of disease at the entry of the nursing home

| □ 1. stroke  □ 2. hypertension  □ 3. diabetes mellitus  □ 4. non-hypertension cardiovascular disease  □ 5. pulmonary disease  □ 6. dementia | □ 7. hepatic disease  □ 8. renal disease  □ 9. cancer  □ 10. degenerative hip joint  □ 11. chronic digestive tract ulcer  □ 12. hyperlipidemia |
| --- | --- |

4. The function at the entry of the nursing home

Cognitive impairment:

| □ 1. no  □ 2. mild | □ 3. moderate  □ 4. severe |
| --- | --- |

Motility:

| □ 1. Free  □ 2. Wheelchair | □ 3. Bedridden |
| --- | --- |

Intake:

| □ 1. by oneself  □ 2. oral feeding | □ 3. gastroenteric tube feeding |
| --- | --- |

Respiratory function:

| □ 1. spontaneous respiration  □ 2. tracheostomy | □ 3. need oxygen |
| --- | --- |

Barth_inex score: _________

(3) Family meeting for palliative care

1. Date of first family meeting: ____/___/______

2. Number of family member participated the first family meeting: ______

3. Number of family meeting: _____________

(4) The information regarding the palliative care:

1. The key family member who signed the DNR

| □ 1. spouse  □ 2. son/daughter | □ 3. siblings  □ 4. others |
| --- | --- |

2. The major diagnosis at the entry of palliative care

| □ 1. terminal cancer  □ 2. amyotrophic lateral sclerosis  □ 3. dementia  □ 4. other cerebral diseases  □ 5. heart failure | □ 6. chronic obstructive pulmonary disease  □ 7. other pulmonary disease  □ 8. chronic liver disease/cirrhosis  □ 9. acute renal failure  □ 10. chronic renal failure/terminal stage of renal disease |
| --- | --- |

3. Other diagnosis at the entry of palliative care

| □ 1. stroke  □ 2. hypertension  □ 3. diabetes mellitus  □ 4. non-hypertension cardiovascular disease  □ 5. pulmonary disease  □ 6. dementia | □ 7. hepatic disease  □ 8. renal disease  □ 9. cancer  □ 10. degenerative hip joint  □ 11. chronic digestive tract ulcer  □ 12. hyperlipidemia |
| --- | --- |

(5) The status at the beginning of the palliative care

1. The date at the beginning of the palliative care: ___/___/_____

2. Cognitive impairment:

| □ 1. no  □ 2. mild | □ 3. moderate  □ 4. severe |
| --- | --- |

Motility:

| □ 1. Free  □ 2. Wheelchair | □ 3. Bedridden |
| --- | --- |

Intake:

| □ 1. by oneself  □ 2. oral feeding | □ 3. gastroenteric tube feeding |
| --- | --- |

Respiratory function:

| □ 1. spontaneous respiration  □ 2. tracheostomy | □ 3. need oxygen |
| --- | --- |

Barth_inex score: _________

(6) The condition within 1 year before receiving the palliative care

1. The date before 1 year of the initial palliative care applied: ____/____/_____

2. Number of department visited per month before palliative care: _______

3. Number and details of medications used per month before palliative care:

□1. Number: _________

□2. Details of medications: ____________________________________

4. Number of admissions within 1 year before palliative care: _______

5. Length of day for the admissions within 1 year before palliative care: _________

6. Admitted to the intensive care unit (ICU) within 1 year before palliative care: □0. no □1. yes

7. Length of day admitted to the intensive care unit (ICU) within 1 year before palliative care: __________

8. Number for ER visits within 1 year before palliative care: ____________

(7) The condition after receiving the palliative care

1. Number and details of medications used per month after palliative care:

□1. Number: _________

□2. Details of medications: ____________________________________

2. Number of department visited per month after palliative care: _______

3. Number of admissions after palliative care: _______

4. Length of day for the admissions after palliative care: _________

5. Admitted to the intensive care unit (ICU) after palliative care: □0. no □1. yes

6. Length of day admitted to the intensive care unit (ICU) after palliative care: __________

7. Number for ER visits after palliative care: ____________

(8) The status of mortality within 180 days after receiving palliative care

1. Death of the subject: □0. no □1. yes

2. Date of Death : ____/____/_______

3. Time of Death: (AM/PM) ____/_____

(9) The information of Family meeting

1. Agreement of the entry of palliative care after family meeting: □0. no □1. yes

2. Number of the family meetings: _______

3. The background of family members

Available spouse: □0. no □1. yes

The number of son/daughter (s): __________

The number of son(s): ________

The number of daughter (s): __________

Number of son/daughter (s) living in Taiwan: _________

Number of son/daughter (s) living in abroad: _________

4. Availability of key family member: □0. no □1. yes

Key family member:

□1. spouse □2. son □3. daughter

□4. siblings □5. others

5. Who participated the family meeting

the spouse: □0. no □1. yes

the number of son/daughter who participated the meeting: _____

the number of other family member who participated the meeting: _____
