# Supplementary material for: Palliative care for nursing home patients with dementia: service evaluation and risk factors of mortality
Source: BMC Palliat Care. 2020 Aug 12;19:122. doi: 10.1186/s12904-020-00627-9 (PMC7425598; doi:10.1186/s12904-020-00627-9)
Supplement: Supplementary file 2 — Additional file 2: e-Figure 1. Study framework. [file 12904_2020_627_MOESM2_ESM.doc]

**Additional file 1.**

e-Figure 1. Study framework
