# Supplementary material for: Palliative care for nursing home patients with dementia: service evaluation and risk factors of mortality
Source: BMC Palliat Care. 2020 Aug 12;19:122. doi: 10.1186/s12904-020-00627-9 (PMC7425598; doi:10.1186/s12904-020-00627-9)
Supplement: Supplementary file 3 — Additional file 3: e-Table 1. Demographic data of the participants upon first receiving institutionalized palliative care. [file 12904_2020_627_MOESM3_ESM.doc]

**Additional file 2.**

e-Table 1. Demographic data of the participants upon first receiving institutionalized palliative care

| Characteristics | Men  (N = 22) | Women  (N = 35) | Statistics^a^ |
| --- | --- | --- | --- |
| **Education** | N (%) | N (%) |  |
| Postgraduate | 1 (4.5) | 0 (0) | P = 0.034 |
| College | 5 (22.7) | 4 (11.4) |  |
| Senior high school | 7 (31.8) | 3 (8.6) |  |
| Junior high school | 3 (13.6) | 4 (11.4) |  |
| Primary school | 5 (22.7) | 11 (31.4) |  |
| Illiterate | 0 (0) | 9 (25.7) |  |
| Unknown | 1 (4.5) | 4 (11.4) |  |
| **Marital** |  |  |  |
| Married | 19 (86.4) | 32 (91.4) | P = 0.171 |
| Unmarried | 3 (13.6) | 1 (2.9) |  |
| Divorced | 0 (0) | 2 (5.7) |  |
| **Couple** |  |  |  |
| Living | 13 (59.1) | 3 (8.6) | P = 0.000 |
| Mortality | 6 (27.3) | 29 (82.9) |  |
| No couple | 3 (13.6) | 3 (8.6) |  |
| **Prior employment** |  |  |  |
| Commercial | 3 (13.6) | 4 (11.4) | P = 0.000 |
| Military | 6 (27.3) | 0 (0) |  |
| Officer | 6 (27.3) | 1 (2.9) |  |
| Housewife | 0 (0) | 24 (68.6) |  |
| Blue-collar | 4 (18.2) | 1 (2.9) |  |
| Education | 0 (0) | 1 (2.9) |  |
| Others | 3 (13.6) | 4 (11.4) |  |
| **Mortality** |  |  |  |
| Survival beyond 180 days after receiving palliative care | 9 (40.9) | 25 (71.4) | P = 0.022 |
| Death within 180 days after receiving palliative care | 13 (59.1) | 10 (28.6) |  |
| **Major Caregiver** |  |  |  |
| Spouse | 4 (18.2) | 0 (0) | P = 0.003 |
| Child | 11 (50.0) | 31 (88.6) |  |
| Siblings | 0 (0) | 1 (2.9) |  |
| Others | 7 (31.8) | 3 (8.6) |  |

^a^Based on the chi-square test
