# Supplementary material for: Palliative care for nursing home patients with dementia: service evaluation and risk factors of mortality
Source: BMC Palliat Care. 2020 Aug 12;19:122. doi: 10.1186/s12904-020-00627-9 (PMC7425598; doi:10.1186/s12904-020-00627-9)
Supplement: Supplementary file 4 — Additional file 4: e-Table 2. Clinical characteristics of the patients upon entry into the nursing home and upon receiving institutionalized palliative care. [file 12904_2020_627_MOESM4_ESM.doc]

**Additional file 3.**

e-Table 2. Clinical characteristics of the patients upon entry into the nursing home and upon receiving institutionalized palliative care

| Characteristic | At the entry of nursing home | Receiving palliative care | Statistics^a^ |
| --- | --- | --- | --- |
|  | (N = 57)  N (%) | (N = 57)  N (%) |  |
| **Co-morbid conditions (multiple choice)** |  |  |  |
| Stroke | 22 (38.6) | 26 (45.6) | - |
| Hypertension | 33 (57.9) | 38 (66.7) | - |
| Diabetes mellitus | 15 (26.3) | 15 (26.3) | - |
| Non-hypertension Cardiovascular disease | 13 (22.8) | 11 (19.3) | - |
| Pulmonary disease | 2 (3.5) | 13 (22.8) | - |
| Renal disease | 3 (5.3) | 3 (5.3) | - |
| Acute renal failure | 3 (5.3) | 6 (10.5) | - |
| Degenerative hip joint (Replacement of hip joint) | 5 (8.8) | 6 (10.5) | - |
| Chronic digestive tract ulcer | 2 (3.5) | 3 (5.3) | - |
| Parkinson disease | 2 (3.5) | 0(0) | - |
| Brain injury | 1 (1.8) | 0(0) | - |
| Hypotension | 1 (1.8) | 0(0) | - |
| Urinary tract infection | 1 (1.8) | 0(0) | - |
| Tracheostomy | 1 (1.8) | 0(0) | - |
| **Cognition** |  |  |  |
| No impairment | 6 (10.5) | 0 (0) | P < 0.001 |
| Mild impairment | 6 (10.5) | 1 (1.8) |  |
| Moderate impairment | 14 (24.6) | 2 (3.5) |  |
| Severe impairment | 31 (54.4) | 54 (94.7) |  |
| **Mobile activity** |  |  |  |
| Free | 10 (17.5) | 0 (0) | P < 0.001 |
| Assistive device | 14 (24.6) | 0 (0) |  |
| Wheelchair | 18 (31.6) | 9 (15.8) |  |
| Bed-ridden | 15 (26.3) | 48 (84.2) |  |
| **Intake** |  |  |  |
| By self | 21 (36.8) | 0 (0) | P < 0.001 |
| Oral feeding | 16 (28.1) | 8 (14.1) |  |
| NG tube feeding | 20 (35.1) | 48 (84.2) |  |
| Intestinal fistula | 0 (0) | 1 (1.8) |  |
| **Urination** |  |  |  |
| By self | 13 (22.8) | 0 (0) | P < 0.001 |
| Diaper | 33 (57.9) | 33 (57.9) |  |
| Urinary tube | 10 (17.5) | 20 (35.1) |  |
| Bladder/intestinal fistula | 1 (1.8) | 4 (7.1) |  |
| **Respiratory function** |  |  |  |
| Tracheostomy | 1 (1.8) | 3 (5.3) | P < 0.001 |
| Spontaneous respiration | 48 (84.2) | 22 (38.6) |  |
| Need oxygen | 8 (14.0) | 32 (56.1) |  |
| Barthel Index, mean/SD | 29.91 (30.16) | 0 (0) | P < 0.001 |
| MMSE, mean (SD) | 7.79 (2.91) | 9.93 (0.37) | P < 0.001 |

MMSE: Mini-Mental State Examination. ^a^Based on a *t* test, chi-square test, or paired *t* test.
